# Bronchopulmonary Dysplasia Predicted by Developing a Machine Learning Model of Genetic and Clinical Information

**DOI:** 10.3389/fgene.2021.689071

**Published:** 2021-07-02

**Authors:** Dan Dai, Huiyao Chen, Xinran Dong, Jinglong Chen, Mei Mei, Yulan Lu, Lin Yang, Bingbing Wu, Yun Cao, Jin Wang, Wenhao Zhou, Liling Qian

**Affiliations:** ^1^Division of Pulmonary Medicine, Children’s Hospital of Fudan University, Shanghai, China; ^2^Molecular Medical Center, Children’s Hospital of Fudan University, Shanghai, China; ^3^Shanghai Key Laboratory of Birth Defects, Shanghai, China; ^4^Department of Neonatology, Children’s Hospital of Fudan University, Shanghai, China

**Keywords:** bronchopulmonary dysplasia, machine learning, prediction model, exome sequencing, premature infants

## Abstract

**Background:**

An early and accurate evaluation of the risk of bronchopulmonary dysplasia (BPD) in premature infants is pivotal in implementing preventive strategies. The risk prediction models nowadays for BPD risk that included only clinical factors but without genetic factors are either too complex without practicability or provide poor-to-moderate discrimination. We aim to identify the role of genetic factors in BPD risk prediction early and accurately.

**Methods:**

Exome sequencing was performed in a cohort of 245 premature infants (gestational age <32 weeks), with 131 BPD infants and 114 infants without BPD as controls. A gene burden test was performed to find risk genes with loss-of-function mutations or missense mutations over-represented in BPD and severe BPD (sBPD) patients, with risk gene sets (RGS) defined as BPD–RGS and sBPD–RGS, respectively. We then developed two predictive models for the risk of BPD and sBPD by integrating patient clinical and genetic features. The performance of the models was evaluated using the area under the receiver operating characteristic curve (AUROC).

**Results:**

Thirty and 21 genes were included in BPD–RGS and sBPD–RGS, respectively. The predictive model for BPD, which combined the BPD–RGS and basic clinical risk factors, showed better discrimination than the model that was only based on basic clinical features (AUROC, 0.915 *vs*. AUROC, 0.814, *P* = 0.013, respectively) in the independent testing dataset. The same was observed in the predictive model for sBPD (AUROC, 0.907 *vs*. AUROC, 0.826; *P* = 0.016).

**Conclusion:**

This study suggests that genetic information contributes to susceptibility to BPD. The predictive model in this study, which combined BPD–RGS with basic clinical risk factors, can thus accurately stratify BPD risk in premature infants.

## Introduction

Bronchopulmonary dysplasia (BPD)—a disorder arising from genetic and environmental risk factors—is one of the most serious complications in premature infants and is responsible for large economic and healthcare burdens. The incidence of BPD in preterm infants [those born with birth weight (BW) between 501 and 1,500 g] varied from 4 to 58.3% in 2003 according to the Vermont Oxford Network data (Payne et al., 2006) and has continued to rise especially among extremely premature infants (Stoll et al., 2010, 2015) due to the improvement in the survival rates of premature infants that benefited from the developments in perinatal medicine and neonatal intensive care. Multiple clinical factors have been implicated in BPD risk, including intrauterine growth restriction (Gortner et al., 1999), low gestational age (GA), low BW, male sex (Korhonen et al., 1999), neonatal respiratory distress syndrome, invasive mechanical ventilation (IMV) (Zhang et al., 2016), sepsis, asphyxia, and—inconsistently—chorioamnionitis (Hartling et al., 2012), race or ethnicity (Rojas et al., 1995; Vesoulis et al., 2020), and mode of delivery (Wang et al., 2014; Chen et al., 2019).

In addition to clinical factors, a considerable amount of research has revealed a genetic basis for BPD and a modulation in the susceptibility of BPD development associated with environmental factors. Genome-wide association studies have identified several single-nucleotide polymorphisms (SNPs) such as rs1245560 in the *SPOCK2* gene of Caucasian and African populations that influence BPD risk (Hadchouel et al., 2011) and other SNPs in SP-A1 (Weber et al., 2000) and SP-B genes (Rova et al., 2004). Numerous rare variants in risk gene sets (RGS) of BPD (BPD–RGS) that are located in pathways related to lung development are also closely associated with the onset and progression of BPD in numerous candidate gene or pathway studies (Carrera et al., 2015; Li et al., 2015; Hadchouel et al., 2020). However, these findings on the genetic etiology of BPD are inconsistent, both reflecting the etiologic molecular heterogeneity of the disease and also showing it to be a complex disease with significant clinical heterogeneity. Despite this, a role for the genetic susceptibility of BPD has been quantified through statistical approaches in two important twin studies (Bhandari, 2006; Lavoie et al., 2008).

Many investigators have carried out research studies in an attempt to predict the development of BPD using clinical factors and respiratory parameters in the neonatal intensive care unit (NICU) of various centers. However, most clinical prediction models are poor to moderate predictors of BPD, e.g., the area under the receiver operating characteristic curve (AUROC) ranged from 0.50 to 0.76 for BPD in an external validation study of a systematic review (Onland et al., 2013). Additionally, the ratio of tidal expiratory flow at 50% of expired volume to peak tidal expiratory flow (as one of the mechanical ventilation parameters) gave an AUROC for the development of moderate/severe BPD (sBPD) of 0.774 (Bentsen et al., 2018); mechanical ventilation at 1 week provided an AUROC for the development of BPD and sBPD of 0.77 and 0.83 (Hunt et al., 2018), respectively; and a multifactorial model that included BW, GA, sex, presence of a hemodynamically significant patent ductus arteriosus (as diagnosed by an echocardiogram), respiratory distress syndrome, hypotension within the first 72 h of life, and intraventricular hemorrhage (IVH) delivered an AUROC for the development of BPD of 0.930—which showed a noticeably improved discriminatory performance, but without favorable clinical maneuverability (Gursoy et al., 2015).

The addition of genetic factors to BPD risk prediction models might improve the ability to accurately predict which infants will develop this significant and serious complication. We therefore developed a risk model for the prediction of BPD and tested the hypothesis that a combined clinical and genetic model that incorporated BPD–RGS would be superior to a clinical-only model.

## Materials and Methods

### Design, Setting, and Participants

We conducted a case–control analysis based on a prospective preterm cohort and consecutively recruited 245 infants from the Children’s Hospital of Fudan University from January 2017 to May 2019 using the following inclusion criteria: premature infants (defined as born with a GA of less than 32 weeks) who required supplemental oxygen on the first day of postnatal life and who were admitted to the NICU. The exclusion criteria were as follows: (1) infants with significant diseases (e.g., major congenital malformations, clinical syndromes, chromosomal abnormalities, systemic infections and shock, or other definite diseases beyond respiratory), (2) refusal for an infant to participate in the study or withdrawal of an infant from intensive care before tracheal extubation was attempted, (3) infants who did not undergo exome sequencing, and (4) infants who died within 7 days after birth.

### Clinical Diagnosis of BPD and Follow-up Collection

**FIGURE 1:**
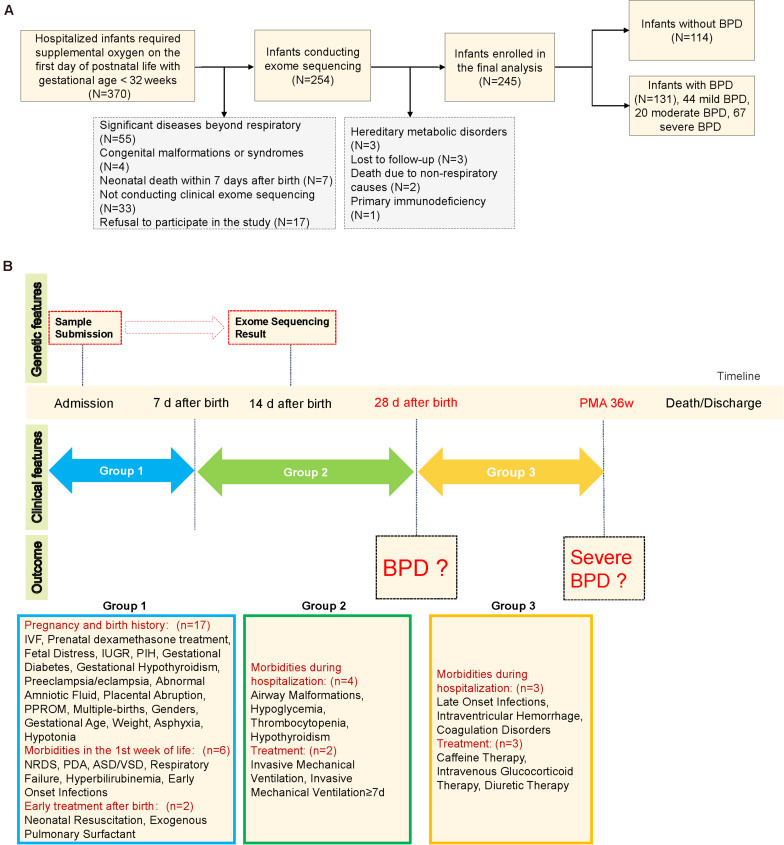
Schematic diagram of the study design. **(A)** Study flow chart. **(B)** Schematic of clinical characteristic collection at each timepoint. Asphyxia was defined as APGAR score of less than seven at 1 min or/and APGAR score of less than seven at 5 min. IVF, *in vitro* fertilization; IUGR, intrauterine growth restriction; PIH, pregnancy-induced hypertension; PPROM, preterm premature rupture of membranes; NRDS, neonatal respiratory distress syndrome; ASD/VSD, atrial septal defect/ventricular septal defect. Patent ductus arteriosus was defined by clinical signs supported by echocardiographic confirmation. Airway malformations: bronchomalacia, tracheomalacia, laryngomalacia, or subglottic stenosis. Early-onset infections: prenatal infection within 72 h of delivery or onset of neonatal pneumonia/sepsis within 7 days of birth. Late-onset infections: infections after 7 days of birth. PMA 36w: postmenstrual age 36 weeks.

Briefly, BPD was diagnosed with respect to a requirement of supplemental oxygen for at least 28 days, with subsequent severity assessment at 36 weeks of postmenstrual age (Jobe and Bancalari, 2001). Perinatal and postnatal information during inpatient hospitalizations and follow-up data were collected until discharge or death, and the timepoints for the collection of clinical characteristics and the corresponding evaluation of outcomes are shown in Figure 1B. Data were doubly entered by two clinical physicians, and the inconsistent data were re-evaluated to reach a consensus.

### Process of Exome Sequencing

Exome sequencing was performed within 7 days after birth for all 245 infants included in the study, and the sequencing results were obtained within 2 weeks after birth. We received a total of 245 infant samples that came from 206 families—including 169 singleton infants, 35 twins, and two triplets. The samples underwent clinical exome sequencing (CES; *n* = 234) and whole-exome sequencing (WES, *n* = 11). In brief, DNA was extracted from peripheral blood specimens using the QIAamp DNA Mini Kit (Qiagen, Pennsylvania, United States) according to the manufacturer’s instructions. We subjected the samples to the Agilent ClearSeq Inherited Disease Kit (for CES) or Agilent Sureselect All Exons Human V5 Kit (for WES) and ran them on the Illumina HiSeq X10 machine, with 150-bp pair-end sequencing. Our detailed sequencing strategy and variant filtration processes were described in detail in our previously published article (Dong et al., 2020). The quality statistics for the 245 samples can be found in Supplementary Materials.

### Variant Not in Unaffected Siblings Analysis

There were 24 infant samples from 11 families (nine twins and two triplets) that each had BPD infants and infants without BPD. For each comparison (BPD *vs*. control or sBPD *vs*. others), we defined a variant in an infant sample as NSV (variant not in unaffected siblings) if it met both of the following criteria: (1) the sample was involved in the case group of the corresponding comparison and (2) the variant did not exist in its control-sibling samples (who were in the control group of the corresponding comparison). Then, for each of the comparisons, we scored a NSVn value for each of the genes as the total number of variants matching the above-mentioned criteria.

### Gene Burden Test

A total of 245 infant samples were used for the gene burden test. For each comparison (BPD *vs*. control or sBPD *vs*. others), we used Fisher’s exact test to compare the number of loss-of-function (LOF) variants (either a frameshift, stop–gain, or canonical splice donor/acceptor variant) and missense variants (MIS) of each gene from cases to those from control subjects. In addition, we also considered the pathogenicity of variants and only selected the variants that were predicted as potentially deleterious variants or deleterious variants by any of the three variant effect prediction tools [SIFT (19561590), PolyPhen-2 (20354512), and MutationTaster (24681721)] for Fisher’s exact test. A threshold of *P* < 0.05 was considered to be statistically significant, and all tests were one-sided (so as to only find genes that are over-represented in the case groups). Thus, each gene produced two separate *P*-values for LOF and MIS.

### Gene Scoring System and Risk Gene Set

For each comparison, we developed a gene scoring system to measure the gene’s contribution by combining the results from the gene burden test and NSV analysis. The score for each gene was defined as follows:

$$\text{Score}=\text{NSVn}+2\times \left(-{\text{log}}_{10}\,\left(P_{\text{LOF}}\right)\right)+\left(-{\text{log}}_{10}\,\left(P_{\text{MIS}}\right)\right).$$

We then defined RGS as the genes with a score greater than two. For two comparisons, the gene set was marked as BPD–RGS (BPD *vs*. control) and sBPD–RGS (sBPD *vs*. others).

The genetic predictors were treated as follows: (1) we extracted genes from the corresponding RGS (BPD–RGS or sBPD–RGS), (2) samples with LOF variants from RGS were scored as 1, and the others were scored as 0 (the feature was recorded as RGS_LOF), and (3) samples with MIS variants from RGS were scored as 1, and the others were scored as 0 (this feature was recorded as RGS_MIS).

### Clinical Predictors

As the clinical diagnoses of BPD and sBPD occur at different time periods with different sets of clinical features available (Figure 1B), we used a total of 31 clinical features (pregnancy and birth history, 17 features; morbidities in the first week of life, six features; early treatment after birth, two features; morbidities during hospitalization, four features; and treatment, two features) for BPD prediction and 37 features for sBPD prediction (morbidities during hospitalization, three features; and treatment, three features). The clinical features were selected based on previous reports or our own experience as likely risk factors for BPD. Missing data were manually imputed by clinical experts. Additionally, we defined the set of basic clinical prediction features with three clinical characteristics—GA, BW, and IMV—as the three most significant predictors for BPD as reported in published studies (Jobe and Bancalari, 2001; Onland et al., 2013).

### Sample Size

According to the previously reported clinical prediction models of BPD, the AUROC is about 0.8 (Onland et al., 2013; Bentsen et al., 2018; Hunt et al., 2018). We hypothesized that adding the genetic information into the prediction model of BPD can improve the AUROC value by 0.1. A required sample size consisting of 103 BPD cases and 103 control cases for a power of 80%, with an alpha value of 0.05, was calculated with PASS, version 11.0. Considering potential loss by insufficient data, we increased the sample size by 15%. We had two tasks: the first one was to predict BPD (cases = 131, controls = 114) and the second was to predict the group of sBPD (cases = 67, controls = 178). We collected 245 samples in this study, which we split into a training dataset (*N* = 172) and testing dataset (*N* = 73) randomly.

### Statistical Analyses

Distributions of continuous variables were evaluated using the Kruskal–Wallis H, and Shapiro–Wilk’s test and Fisher’s exact probability test were used to compare the differences in categorical variables. Data are expressed as medians and interquartile ranges (Q1–Q3) for non-parametric distributions, and categorical data are expressed as numbers and percentages. We clustered clinical characteristics by the Ward agglomeration method, and distance was calculated using the Canberra method. The number of clusters was specified as three. A multivariable logistic regression analysis was used to assess independent associations between clinical characteristics and BPD or sBPD, adjusting for GA and BW in 245 infants. The odds ratios (ORs) and 95% confidence intervals were estimated.

We constructed three predictive models for both BPD and sBPD by using different sets of predictors as follows: (1) the set of three basic clinical features (GA, BW, and history of IMV), (2) three basic clinical features with two genetic features (RGS_LOF and RGS_MIS), and (3) all clinical features selected by LASSO (R package: glmnet, 15 features for BPD prediction and 19 for sBPD prediction, Supplementary Figure 1) with two genetic features (RGS_LOF and RGS_MIS).

For each predictive model, we applied LASSO (R package: glmnet) to generate the predictive model on the training dataset. A 10-fold cross-validation was performed to select the optimal lambda (penalty for the number of characteristics), which determined the performance of the lasso model (number of features included in the model and predictive deviations). We then evaluated the predictive performance for the three lasso models in independent testing datasets. The area under the receiver operating characteristic curve (AUROC) was used for model evaluation, and the statistical differences between models were tested using Delong’s test. We performed all analyses using R software (version 3.6.0,^1^).

### Ethics and Informed Consent

This study was approved by the Ethics Committee of the Children’s Hospital of Fudan University (no. 2016-97), and informed consent for DNA analysis of peripheral blood cells was obtained from the study participants in accordance with the time of collection.

## Results

### Patient Cohort of Preterm Infants

A consecutively enrolled total of 370 premature infants with a GA of <32 weeks were admitted to our NICU during the study period. One hundred sixteen infants were ruled out by the exclusion criteria, and nine (four for clear etiology, three lost to follow-up, and two died of non-respiratory causes) were excluded from further analysis; ultimately, 245 infants were included in the final analysis (Figure 1A). There were 131 (53.5%) infants diagnosed with BPD, including 67 (51.1%) with sBPD (including nine infants who died due to respiratory dysfunction before postmenstrual age 36 weeks), 20 (15.3%) with moderate BPD, and 44 (33.6%) with mild BPD. The infants with BPD exhibited lower GA (28.1 *vs*. 30 weeks, *P* < 0.001) and BW (1,090 *vs*. 1,332.5 g, *P* < 0.001) compared to infants with no BPD. Furthermore, the BPD infants manifested a higher rate of IMV (77.9 *vs*. 35.1%, *P* < 0.001), IMV ≥7 days (55 *vs*. 6.1%, *P* < 0.001), early-onset infections (<7 days) (87 *vs*. 70.2%, *P* = 0.002), late-onset infections (≥7 days) (45.8 *vs*. 27.2%, *P* = 0.004), grades III and IV IVH (42 *vs*. 17.5%, *P* < 0.001), and death (14.5 *vs*. 0, *P* < 0.001, Table 1). We noted that nine of the 19 infants who died before the evaluation of BPD severity had been assigned to the sBPD group. Other clinical characteristics are depicted in Supplementary Table 2.

**TABLE 1:** Clinical characteristics of 245 premature infants.

**Characteristic**	**Total infants (*n* = 245)**	**BPD (*n* = 131)**	**No BPD (*n* = 114)**	***P*-value**	**Mild BPD (*n* = 44)**	**Moderate BPD (*n* = 20)**	**Severe BPD (*n* = 67)**	***P*-value**
Gestational age [week, median (IQR)]	29.1 (27.9–30.6)	28.1 (27.1–29.8)	30 (28.6–31.1)	<0.001	29 (27.8–29.7)	28.8 (27.5–30.6)	27.6 (26.6–29.4)	<0.001
Birth weight [g, median (IQR)]	1,200 (1,020–1,410)	1,090 (945–1,260)	1,332.5 (1,131.3–1,570)	<0.001	1,160 (1,072.5–1,280)	1,110 (1,017.5–1,271.3)	1,035 (915–1,200)	<0.001
Sex (male), no. (%)	140 (57.1%)	80 (61.1%)	60 (52.6%)	0.229	26 (59.1%)	13 (65%)	41 (61.2%)	0.579
Multiple births, no. (%)	117 (47.8%)	72 (55%)	45 (39.5%)	0.022	27 (61.4%)	13 (65%)	32 (47.8%)	0.032
Neonatal respiratory distress syndrome, no. (%)	194 (79.2%)	113 (86.3%)	81 (71.1%)	0.006	40 (90.9%)	18 (90%)	55 (82.1%)	0.019
Respiratory failure, no. (%)	229 (93.5%)	131 (100%)	98 (86%)	<0.001	44 (100%)	20 (100%)	67 (100%)	<0.001
Atrial septal defect or ventricular septal defect, no. (%)	63 (25.7%)	41 (31.3%)	22 (19.3%)	0.046	6 (13.6%)	9 (45%)	26 (38.8%)	0.001
Patent ductus arteriosus, no. (%)	205 (83.7%)	118 (90.1%)	87 (76.3%)	0.006	40 (90.9%)	19 (95%)	59 (88.1%)	0.029
Thrombocytopenia, no. (%)	27 (11%)	23 (17.6%)	4 (3.5%)	0.001	2 (4.5%)	4 (20%)	17 (25.4%)	<0.001
Coagulation disorders, no. (%)	73 (29.8%)	52 (39.7%)	21 (18.4%)	<0.001	10 (22.7%)	9 (45%)	33 (49.3%)	<0.001
Intraventricular hemorrhage (grades III and IV), no. (%)	75 (30.6%)	55 (42%)	20 (17.5%)	<0.001	13 (29.5%)	10 (50%)	32 (47.8%)	<0.001
Early-onset infections (<7 days), no. (%)	194 (79.2%)	114 (87%)	80 (70.2%)	0.002	35 (79.5%)	16 (80%)	63 (94%)	0.002
Late-onset infections (≥7 days), no. (%)	91 (37.1%)	60 (45.8%)	31 (27.2%)	0.004	14 (31.8%)	13 (65%)	33 (49.3%)	0.001
Airway malformations, no. (%)	11 (4.5%)	10 (7.6%)	1 (0.9%)	0.025	1 (2.3%)	1 (5%)	8 (11.9%)	0.005
Invasive mechanical ventilation (IMV), no. (%)	142 (58%)	102 (77.9%)	40 (35.1%)	<0.001	24 (54.5%)	17 (85%)	61 (91%)	<0.001
IMV ≥7 days, no. (%)	79 (32.2%)	72 (55%)	7 (6.1%)	<0.001	10 (22.7%)	11 (55%)	51 (76.1%)	<0.001
Exogenous pulmonary surfactant, no. (%)	170 (69.4%)	102 (77.9%)	68 (59.6%)	0.003	32 (72.7%)	15 (75%)	55 (82.1%)	0.013
Intravenous glucocorticoid therapy, no. (%)	26 (10.6%)	25 (19.1%)	1 (0.9%)	<0.001	2 (4.5%)	1 (5%)	22 (32.8%)	<0.001
Caffeine therapy, no. (%)	197 (80.4%)	116 (88.5%)	81 (71.1%)	0.001	40 (90.9%)	19 (95%)	57 (85.1%)	0.005
Death, no. (%)	19 (7.8%)	19 (14.5%)	0	<0.001	0	0	19 (28.4%)	<0.001

*All summary data are medians (25–75% percentile) or counts (%).*

*wk, week; gr, gram.*

### Clinical Risk Factors for BPD and Severity of BPD in Preterm Infants

**FIGURE 2:**
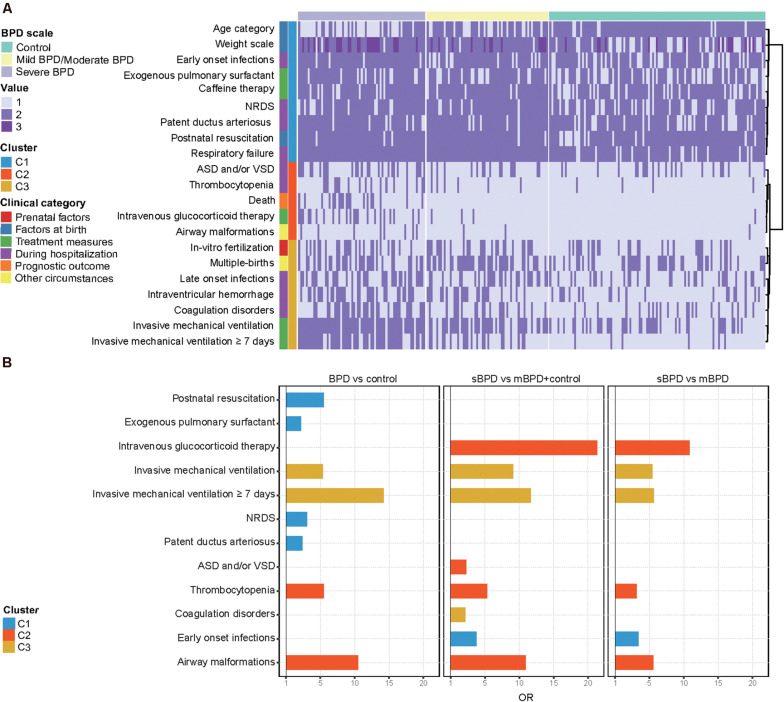
Distribution of infant clinical characteristics. **(A)** Cluster analysis of clinical characteristics of all infants. **(B)** Significant clinical characteristics in three comparisons. Twenty-one clinical characteristics that significantly differed between bronchopulmonary dysplasia (BPD) and controls or between any two BPD levels (*p* < 0.05) were selected. Weight scale including low BW (1,500–2,499 g), very low BW (1,000–1,499 g), and extremely low BW (<1,000 g) were represented by values 1–3, respectively. Age category including extremely preterm (<28 weeks) and very preterm (28 to 32 weeks) was represented by values 1 and 2, respectively. ASD/VSD, atrial septal defect/ventricular septal defects; NRDS, neonatal respiratory distress syndrome; sBPD, severe BPD; mBPD, mild and moderate BPD.

Twenty-one clinical characteristics that significantly differed between BPD and controls or between any two BPD levels (*p* < 0.05) were selected. We grouped these clinical features into three clusters according to their co-existence with sBPD and mBPD (mild and moderate BPD), as shown in Figure 2A. Generally, Cluster1 features were related to the general situation at birth and early complications within 7 days after birth. Congenital airway anomaly and congenital heart disease were principally shown in Cluster2 features. Cluster3 features were primarily involved in late complications beyond 7 days after birth and included a condition of IMV. We then validated the independent contribution to the clinical features between case and control groups in three comparisons using multivariable logistic regression and adjusting for GA and BW. The OR for each feature in each of the comparisons is shown in Figure 2B. IMV ≥7 days and airway malformations were both the top two clinical factors for BPD (OR = 14.209, 95% CI, 6.102–38.112 and OR = 10.485, 95% CI, 1.739–202.955, respectively) and sBPD development (OR = 11.686, 95% CI, 5.724–24.867 and OR = 10.954, 95% CI, 2.604–58.863, respectively). Furthermore, the need for intravenous glucocorticoid therapy was higher in infants with sBPD (OR = 21.308, 95% CI, 6.792–84.288) relative to the other groups. Atrial septal defects and/or ventricular septal defects and coagulation disorders were risk factors for sBPD compared to other groups, but not in sBPD *vs*. mBPD comparison.

### Risk Genes for BPD and Severe BPD

A total of 30 BPD–RGS and 21 sBPD–RGS (with a score of significant genes >2) were identified based on the gene burden test and NSV analysis in the comparison of BPD *vs*. control and sBPD *vs*. others, respectively (Table 2 and Supplementary Table 2). Most of these genes (such as *OBSL1*, *GNAS*, *TCIRG1*, and *C5*) are involved in susceptibility to infection and inflammatory response, cellular and immune regulation, cellular biologic function, and metabolic biologic processes closely related to early development and organogenesis, which are important in the occurrence of biologic dysfunction in BPD development (Supplementary Figure 2). In addition, there were 16 (53.3%) and eight (38.1%) genes that also appeared in the set of significant genes, respectively, when using potentially deleterious variants or deleterious variants for the burden test (Supplementary Table 3). The RGS obtained in the study showed a very little overlap with other reported studies and was similar to that for studies that supported a large genetic heterogeneity (Supplementary Figure 3).

**TABLE 2:** Genes with a significant burden for LOF/MIS variants.

**Comparison**	**Gene**	**Samples**	**Case (*n*) LOF**	**Case (*n*) MIS**	**Control (*n*) LOF**	**Control (*n*) MIS**	**Control (*n*)**	**Case (*n*)**	**LOF *P*-value**	**MIS *P*-value**	**NSV (*n*)**	**Score**
BPD *vs*. control	OBSL1	31	11	18	0	13	114	131	<0.001	0.362	1	7.600
BPD *vs*. control	NTRK1	34	1	15	1	2	114	131	0.785	0.002	2	4.884
BPD *vs*. control	CHRNA4	10	0	10	1	0	114	131	1.000	0.002	1	3.791
BPD *vs*. control	PDE11A	24	7	14	2	3	114	131	0.124	0.011	0	3.766
BPD *vs*. control	FRG1	24	9	10	2	3	114	131	0.049	0.070	0	3.764
BPD *vs*. control	SPTAN1	26	0	19	0	4	114	131	1.000	0.002	1	3.613
BPD *vs*. control	DCC	17	1	12	0	2	114	131	0.535	0.011	1	3.507
BPD *vs*. control	BDP1	14	3	10	0	2	114	131	0.151	0.030	0	3.159
BPD *vs*. control	C5	10	1	7	1	0	114	131	0.785	0.012	1	3.147
sBPD *vs*. mBPD/control	ACADSB	10	5	1	3	6	178	67	0.037	0.897	2	4.907
sBPD *vs*. mBPD/control	TCIRG1	15	3	5	0	13	178	67	0.020	0.578	1	4.646
sBPD *vs*. mBPD/control	OBSL1	31	7	10	4	21	178	67	0.011	0.323	0	4.408
sBPD *vs*. mBPD/control	FGFR3	17	0	12	0	9	178	67	1.000	0.003	1	3.594
sBPD *vs*. mBPD/control	BDP1	14	3	2	0	10	178	67	0.020	0.887	0	3.459
sBPD *vs*. mBPD/control	RBBP8	9	0	7	0	3	178	67	1.000	0.005	1	3.290
sBPD *vs*. mBPD/control	SPG7	20	0	8	1	5	178	67	1.000	0.008	1	3.077
sBPD *vs*. mBPD/control	GNAS	17	0	11	0	6	178	67	1.000	<0.001	0	3.028

*LOF/MIS, loss-of-function variants and missense variants; sBPD, severe bronchopulmonary dysplasia; mBPD, mild and moderate bronchopulmonary dysplasia; NSV, variant not in unaffected siblings.*

### Machine Learning Model Generation and Testing for the Prediction of BPD or Severe BPD

**FIGURE 3:**
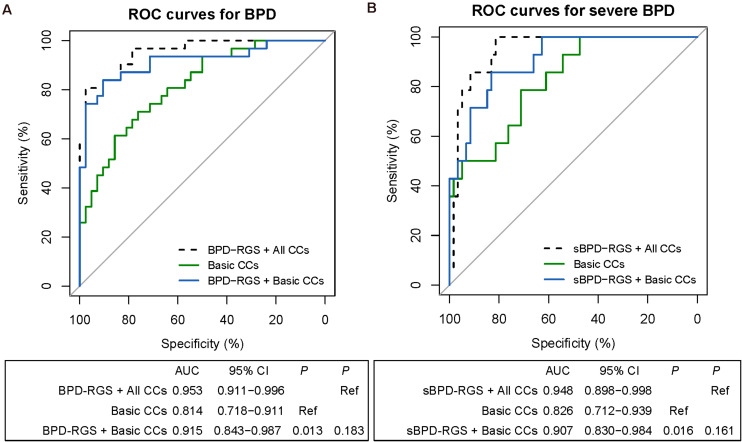
Receiver operating characteristic (ROC) analyses of predictive models for infants with bronchopulmonary dysplasia (BPD) or severe BPD (sBPD). The comparisons of predictive models for BPD and sBPD. *P*-values show the areas under the ROC curves (AUROCs) between the different models. Clinical characteristics include the variables in a separate lasso model (Supplementary Figure 1). **(A)** ROC analyses of the prediction of BPD by the combination of BPD–risk gene set (RGS) and all clinical characteristic model, the basic clinical characteristic model, and the combined BPD–RGS and basic clinical characteristics model. **(B)** ROC analyses of the prediction of severe BPD by the combined sBPD–RGS and all clinical characteristic model, the basic clinical characteristic model, and the combined sBPD–RGS and basic clinical characteristics model. CCs, clinical characteristics.

Herein we integrated clinical and genetic features to predict the risk of BPD and sBPD by using a machine learning model. Different sets of clinical features were used for each task (Figure 1B) in combination with two genetic predictors (BPD–RGS or sBPD–RGS for LOF and MIS), and ROC analysis was used to evaluate the performance of the predictive models. We obtained an excellent predictive result using basic clinical characteristics combined with BPD–RGS (AUROC of 0.915, 0.843–0.987) compared with only using basic clinical characteristics (AUROC of 0.814, 0.718–0.911) (*P* = 0.013), and the results were similar when using all clinical characteristics combined with the BPD–RGS variant burden (AUROC, 0.953; 0.911–0.996) (*P* = 0.183; Figure 3A). For the sBPD prediction model, the model results revealed a higher AUROC value (0.907; 0.830–0.984) using basic clinical characteristics combined with the BPD–RGS variant burden compared with using basic clinical characteristics (0.826, 0.712–0.939) (*P* = 0.016; Figure 3B). Surprisingly, when we built prediction models using the associated genes found by potentially deleterious variants or deleterious variants, we did not find a significant improvement in the accuracy of the two models (model BPD: AUROC, 0.872 *vs*. AUROC, 0.814, *P* = 0.125; model sBPD: AUROC, 0.881 *vs*. AUROC, 0.827, *P* = 0.32) (Supplementary Figure 4). Additionally, we found that genetic factors also contributed to the prediction model with respect to deaths in infants with BPD (AUROC, 0.891 *vs*. AUROC, 0.859, *P* = 0.258) (Supplementary Figure 5); however, we posit that its predictive power was not robust due to the limited number of samples from deceased infants, and more evidence in a larger cohort is therefore needed to support this finding.

## Discussion

Bronchopulmonary dysplasia remains the most common long-term respiratory morbidity affecting prematurely born infants and has been a significant burden on families and public healthcare resources. Its multi-factorial pathogenesis arises from a complex interaction between genetic and environmental influences, and a plethora of studies are devoted to the early prediction of the development of BPD as well as the severity of BPD and poor prognosis. Clinical characteristics such as GA, BW, race and ethnicity, gender, APGAR score, and amount of oxygen administered/positive inspiratory pressure/mean airway pressure are the common predictors in a variety of predictive models described in previous studies. Despite these efforts, no reliable and reproducible risk stratification model has been found that would allow an early diagnosis and ease of application within NICUs. A single predictive factor cannot accurately predict the BPD outcome because BPD is a multifactorial disease, and therefore current multivariate modeling demonstrates that the greater the number of clinical predictor variables, the better the modeling results tend to be (Laughon et al., 2011; Gursoy et al., 2015). However, this also increases complexity and impairs practicality in clinical practice. In our cohort of premature infants, we found that low BW, low GA, IMV, IMV ≥7 days, IVH, and early- and/or late-onset infections were risk factors for BPD—and this was also demonstrated in numerous other studies (Lapcharoensap et al., 2015). In addition, we uncovered a higher proportion of intravenous glucocorticoid therapy use in infants with sBPD, which may be explained by the severe fundamental lung conditions observed in infants who later developed sBPD.

That there is a significant contribution of genetic factors to the development of BPD is well established, and considerable research effort has been directed toward elucidating the genetic etiologies and pathogenic genetic mechanisms involved. Although these studies yielded inconsistent and sometimes controversial results in different cohorts due to the vast heterogeneity of infants with BPD, they still provided very valuable knowledge and increased our understanding of the nature of BPD development at the DNA level. To overcome this dilemma, several studies have already focused on the genetic basis for BPD in infants with extreme phenotypes of early respiratory morbidity and infants who exhibited a good response to certain therapies (Hamvas et al., 2018; Torgerson et al., 2018). Advancements in the care of patients with BPD over the next decades are dependent upon improved understanding and the use of disease phenotyping in infants with BPD to enable better risk stratification and targeted therapeutic interventions. Thus, to better predict BPD development and the severity of BPD—and to identify those infants at greatest risk for poor outcomes—there is a need to identify perinatal risk factors on the basis of genetic predisposition.

Unfortunately, there are a few studies that focus on genetic aspects combined with clinical risk factors in the prediction of BPD development. In the present study, we developed a scoring system to identify the gene burden in infants with BPD and obtained BPD–RGS (with scores >2) with most of the genes tightly correlated with drug metabolism, immune response, and organ development as described in the functional enrichment analysis—and thus playing essential roles in the proper development and function of the postnatal lung. We further constructed predictive models of BPD development using BPD–RGS combined with clinical factors; intriguingly, the use of gene burdens resulted in a significant increase of 10% in the AUROC of the model using only basic clinical characteristics (GA, BW, and IMV) for BPD and 8% for sBPD. These results suggested that we can classify the probable status of the disease in admitted patients using a small amount of clinical information and the patients’ BPD–RGS sequencing information and that this can then be further validated in a larger independent cohort. However, the findings of significant improvement in the accuracy of the two predictive models mentioned above are no longer present when prediction models were built using the associated genes found by potentially deleterious or deleterious variants, suggesting that some variants of undetermined significance may also have certain effects on protein function especially considering the overall consequences of multiple variants.

Our findings would allow an early and accurate identification of infants who could potentially benefit from focused therapy and would take a significant step forward in the comprehensive and personalized care of individuals with BPD. However, despite our encouraging results, we still need to accrue additional genetic study cohorts or enlarge our present cohort to develop a pathway scoring system that allows the progression of BPD–RGS into the pathway level, overcoming and stratifying the tremendous genetic heterogeneity that is present.

Some limitations to this study should also be acknowledged. A single-center design leads to missing data and unavoidable biases in identifying and recruiting participants. Further validation beyond this initial exploratory cohort is warranted. In order to demonstrate the robustness of the results, we intend to further verify this predictive model in another external cohort (currently recruiting). Despite these limitations, this study was the first one designed to combine clinical factors and genetic variations so as to predict BPD occurrence and severity. Thus, our model might assist clinicians in the earlier diagnosis of BPD, guide clinical therapy and prognosis, allow for appropriate decision-making, and optimize the use of hospital resources.

## Conclusion

We first determined the genetic contribution in predictive models of BPD development and showed that three basic clinical characteristics combined with BPD–RGS achieved a high prediction accuracy of models that predicted BPD development and its severity.

## Data Availability Statement

The datasets presented in this study can be found in online repositories. The names of the repository/repositories and accession number(s) can be found in the article/Supplementary Material.

## Ethics Statement

The studies involving human participants were reviewed and approved by the Ethics Committee of the Children’s Hospital of Fudan University (approval number: 2016-97). Written informed consent to participate in this study was provided by the participants’ legal guardian/next of kin. Informed consent was obtained from the parents of patients who were recruited in the present study.

## Author Contributions

LQ and WZ contributed to research conception and design and take responsibility for the accuracy and integrity of the data analysis. YC, JW, and MM contributed to the patient enrollment, sample collection, and follow-up. BW, YL, LY, and XD contributed to the genetic testing and bioinformatics analysis and drafted the report. DD, HC, and JC contributed to the data acquisition, analysis, and interpretation. DD and HC contributed to the drafting of the manuscript. LQ contributed to the critical revision of the manuscript. All authors approved the final manuscript and had full access to all data in the study.

## Conflict of Interest

The authors declare that the research was conducted in the absence of any commercial or financial relationships that could be construed as a potential conflict of interest.

## Footnotes

1 http://cran.r-project.org

## Abbreviations

ASD: atrial septal defects
AUROC: area under the curve
AUROC: area under the receiver operating characteristic curve
BW: birth weight
BPD: bronchopulmonary dysplasia
CES: clinical exome sequencing
NSV: variant not in unaffected siblings
GA: gestational age
IMV: invasive mechanical ventilation
IUGR: intrauterine growth restriction
IVH: intraventricular hemorrhage
LOF: loss-of-function variants
MIS: missense variants
NICU: neonatal intensive care unit
NRDS: neonatal respiratory distress syndrome
RGS: risk gene sets
sBPD: severe bronchopulmonary dysplasia
SNPs: single-nucleotide polymorphisms
VSD: ventricular septal defects
WES: whole-exome sequencing.
