# Supplementary material for: Bronchopulmonary Dysplasia Predicted by Developing a Machine Learning Model of Genetic and Clinical Information
Source: Front Genet. 2021 Jul 2;12:689071. doi: 10.3389/fgene.2021.689071 (PMC8283015; doi:10.3389/fgene.2021.689071)
Supplement: Supplementary file 1 [file Data_Sheet_1.pdf]

Bronchopulmonary dysplasia predicted by developing a machine-learning model of genetic and clinical information

Dan Dai[+], Huiyao Chen[+], Xinran Dong, Jinglong Chen, Mei Mei, Yulan Lu, Lin Yang, Bingbing Wu, Yun Cao, Jin Wang, Wenhao Zhou[*], Liling Qian[*]
+These authors contributed equally to this work.
*Corresponding co-senior authors.

Online Data Supplement

Quality assessment for sequencing data used in this study

Short-read exome sequencing:

There are 234 samples performed CES (clinical exome sequencing) and 11 samples performed WES (whole-exome sequencing). In brief, DNA was extracted from peripheral blood specimens using the QIAamp DNA Mini Kit (Qiagen, Pennsylvania, USA) according to the manufacturer's instructions. The samples were performed using Agilent ClearSeq Inherited Disease Kit (CES) or Agilent Sureselect All Exons Human V5 Kit (WES), run on the Illumina HiSeq X10, with 150 bp pair-end sequencing. The sequencing quality statistics of the 245 samples were as follows:

	**CES**	**WES**
Mean depth of target region (X)	221.99	118.8
Reads mapping rate (%)	99.85%	99.88%
Fraction of target covered ≥ 20x	99.13%	97.14%
On-target rate (%)	77.25%	81.36%
Total Reads (M)	27.72	73.51
Total effective data (Gb)	4.14	10.85
Rate of nucleotide mismatch (%)	0.70%	0.37%

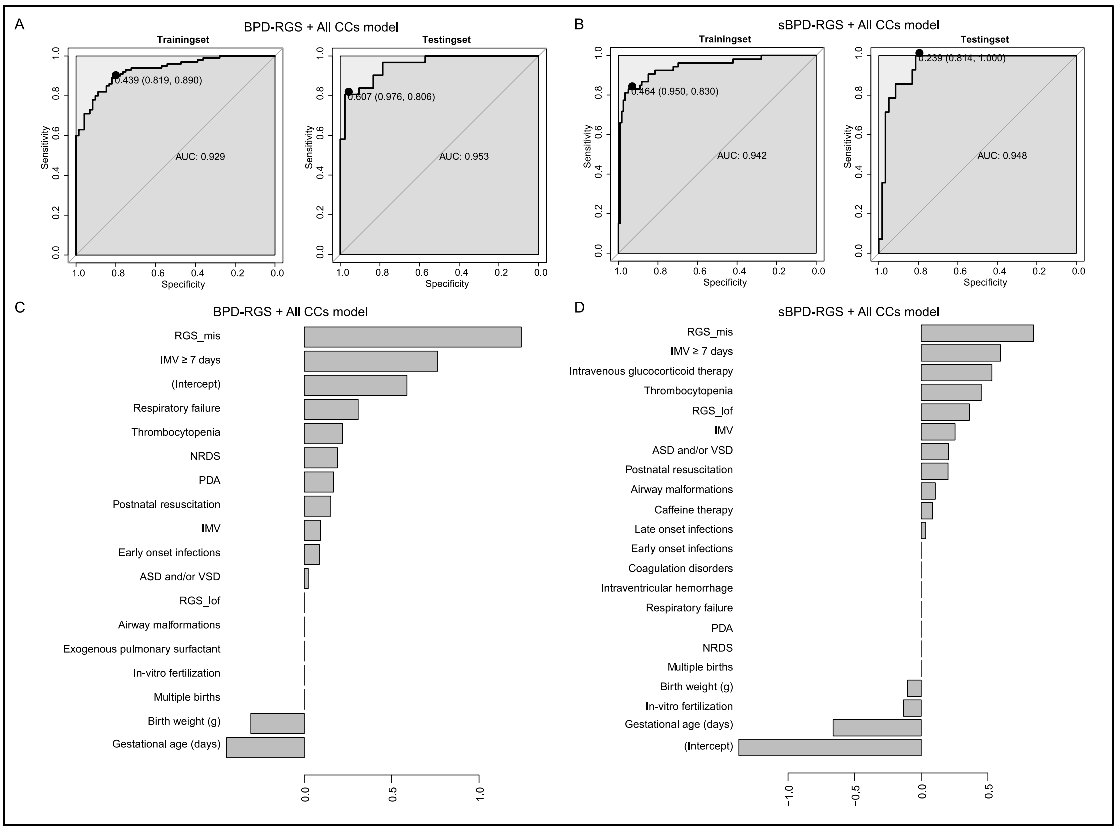

e-figure 1: Feature's Coefficient in Predictive Models for BPD and Severe BPD.

(A) The areas under the ROC curves (AUCs) of the combined BPD-RGS and all clinical

characteristics model in the training set and test set. (B) The areas under the ROC curves (AUCs) of the combined sBPD-RGS and all clinical-characteristics model in the training set and test set. (C) All clinical characteristics and BPD-RGS as selected by the lasso model and their coefficients. (D) All clinical characteristics and sBPD-RGS as selected by the lasso model and their coefficients.

CCs: clinical characteristics; RGS: risk gene set; IMV: invasive mechanical ventilation; NRDS: neonatal respiratory distress syndrome; PDA: Patent ductus arteriosus; ASD/VSD: Atrial septal defect/ventricular septal defect.

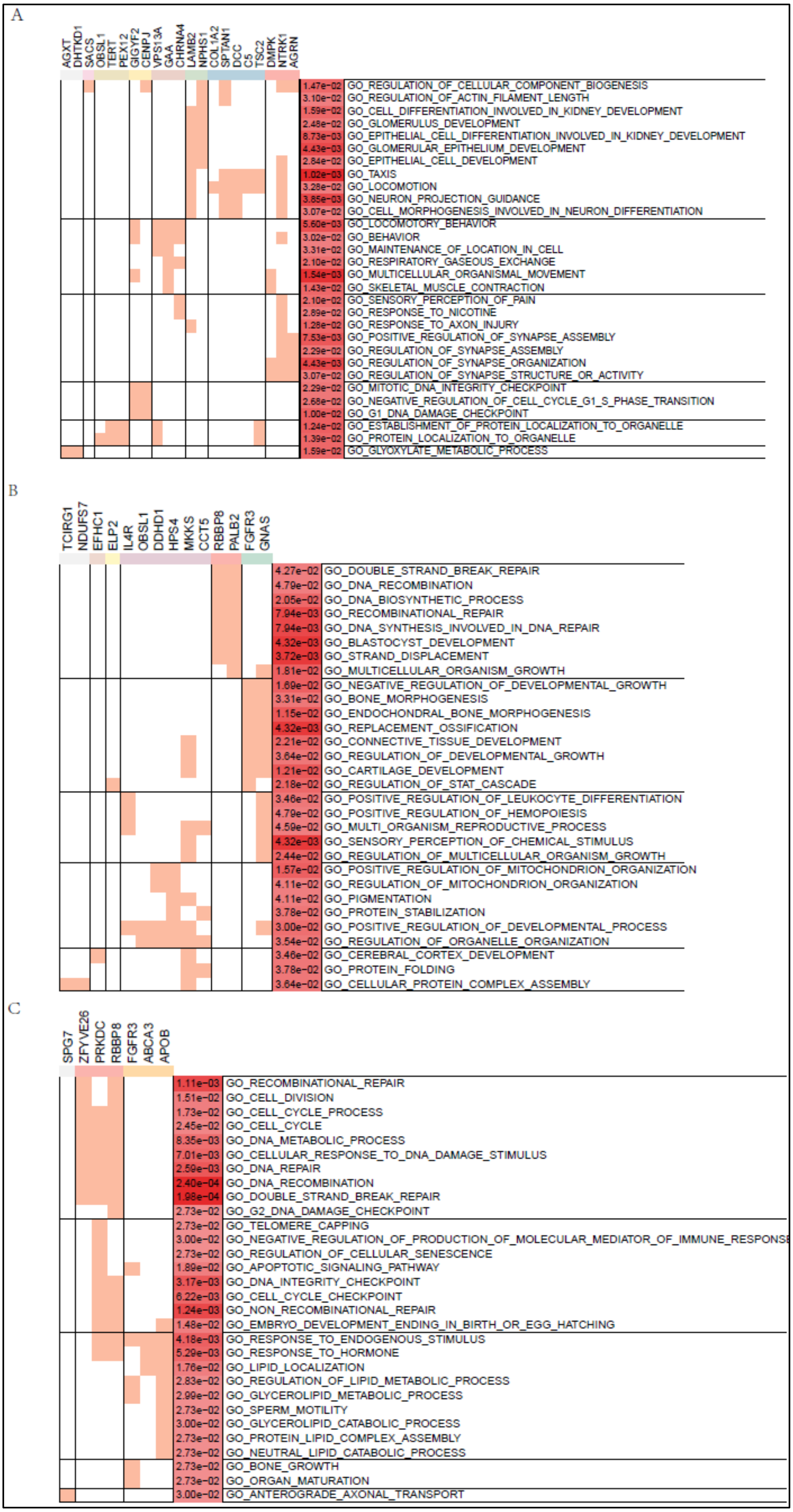
A
AGXT
DHTKD1
SACS
OBSL1
TERT
PEX12
GIGYF2
CENPJ
VPS13A
GAA
CHRNA4
LAMB2
NPHS1
COL1A2
SPTAN1
DCC
C5
TSC2
DMPK
NTRK1
AGRN
1.47e-02 GO_REGULATION_OF_CELLULAR_COMPONENT_BIOGENESIS
3.10e-02 GO_REGULATION_OF_ACTIN_FILAMENT_LENGTH
1.59e-02 GO_CELL_DIFFERENTIATION_INVOLVED_IN_KIDNEY_DEVELOPMENT
2.48e-02 GO_GLOMERULUS_DEVELOPMENT
8.73e-03 GO_EPITHELIAL_CELL_DIFFERENTIATION_INVOLVED_IN_KIDNEY_DEVELOPMENT
4.43e-03 GO_GLOMERULAR_EPITHELIUM_DEVELOPMENT
2.84e-02 GO_EPITHELIAL_CELL_DEVELOPMENT
1.02e-03 GO_TAXIS
3.28e-02 GO_LOCOMOTION
3.85e-03 GO_NEURON_PROJECTION_GUIDANCE
3.07e-02 GO_CELL_MORPHOGENESIS_INVOLVED_IN_NEURON_DIFFERENTIATION
5.60e-03 GO_LOCOMOTORY_BEHAVIOR
3.02e-02 GO_BEHAVIOR
3.31e-02 GO_MAINTENANCE_OF_LOCATION_IN_CELL
2.10e-02 GO_RESPIRATORY_GASEOUS_EXCHANGE
1.54e-03 GO_MULTICELLULAR_ORGANISMAL_MOVEMENT
1.43e-02 GO_SKELETAL_MUSCLE_CONTRACTION
2.10e-02 GO_SENSORY_PERCEPTION_OF_PAIN
2.89e-02 GO_RESPONSE_TO_NICOTINE
1.28e-02 GO_RESPONSE_TO_AXON_INJURY
7.53e-03 GO_POSITIVE_REGULATION_OF_SYNAPSE_ASSEMBLY
2.29e-02 GO_REGULATION_OF_SYNAPSE_ASSEMBLY
4.43e-03 GO_REGULATION_OF_SYNAPSE_ORGANIZATION
3.07e-02 GO_REGULATION_OF_SYNAPSE_STRUCTURE_OR_ACTIVITY
2.29e-02 GO_MITOTIC_DNA_INTEGRITY_CHECKPOINT
2.68e-02 GO_NEGATIVE_REGULATION_OF_CELL_CYCLE_G1_S_PHASE_TRANSITION
1.00e-02 GO_G1_DNA_DAMAGE_CHECKPOINT
1.24e-02 GO_ESTABLISHMENT_OF_PROTEIN_LOCALIZATION_TO_ORGANELLE
1.39e-02 GO_PROTEIN_LOCALIZATION_TO_ORGANELLE
1.59e-02 GO_GLYOXYLATE_METABOLIC_PROCESS
B
TCIRG1
NDUFS7
EFHC1
ELP2
IL4R
OBSL1
DDHD1
HPS4
MKKS
CCT5
RBBP8
PALB2
FGFR3
GNAS
4.27e-02 GO_DOUBLE_STRAND_BREAK_REPAIR
4.79e-02 GO_DNA_RECOMBINATION
2.05e-02 GO_DNA_BIOSYNTHETIC_PROCESS
7.94e-03 GO_RECOMBINATIONAL_REPAIR
7.94e-03 GO_DNA_SYNTHESIS_INVOLVED_IN_DNA_REPAIR
4.32e-03 GO_BLASTOCYST_DEVELOPMENT
3.72e-03 GO_STRAND_DISPLACEMENT
1.81e-02 GO_MULTICELLULAR_ORGANISM_GROWTH
1.69e-02 GO_NEGATIVE_REGULATION_OF_DEVELOPMENTAL_GROWTH
3.31e-02 GO_BONE_MORPHOGENESIS
1.15e-02 GO_ENDOCHONDRAL_BONE_MORPHOGENESIS
4.32e-03 GO_REPLACEMENT_OSSIFICATION
2.21e-02 GO_CONNECTIVE_TISSUE_DEVELOPMENT
3.64e-02 GO_REGULATION_OF_DEVELOPMENTAL_GROWTH
1.21e-02 GO_CARTILAGE_DEVELOPMENT
2.18e-02 GO_REGULATION_OF_STAT_CASCADE
3.46e-02 GO_POSITIVE_REGULATION_OF_LEUKOCYTE_DIFFERENTIATION
4.79e-02 GO_POSITIVE_REGULATION_OF_HEMOPOIESIS
4.59e-02 GO_MULTI_ORGANISM_REPRODUCTIVE_PROCESS
4.32e-03 GO_SENSORY_PERCEPTION_OF_CHEMICAL_STIMULUS
2.44e-02 GO_REGULATION_OF_MULTICELLULAR_ORGANISM_GROWTH
1.57e-02 GO_POSITIVE_REGULATION_OF_MITOCHONDRION_ORGANIZATION
4.11e-02 GO_REGULATION_OF_MITOCHONDRION_ORGANIZATION
4.11e-02 GO_PIGMENTATION
3.78e-02 GO_PROTEIN_STABILIZATION
3.00e-02 GO_POSITIVE_REGULATION_OF_DEVELOPMENTAL_PROCESS
3.54e-02 GO_REGULATION_OF_ORGANELLE_ORGANIZATION
3.46e-02 GO_CEREBRAL_CORTEX_DEVELOPMENT
3.78e-02 GO_PROTEIN_FOLDING
3.64e-02 GO_CELLULAR_PROTEIN_COMPLEX_ASSEMBLY
C
SPG7
ZFYVE26
PRKDC
RBBP8
FGFR3
ABCA3
APOB
1.11e-03 GO_RECOMBINATIONAL_REPAIR
1.51e-02 GO_CELL_DIVISION
1.73e-02 GO_CELL_CYCLE_PROCESS
2.45e-02 GO_CELL_CYCLE
8.35e-03 GO_DNA_METABOLIC_PROCESS
7.01e-03 GO_CELLULAR_RESPONSE_TO_DNA_DAMAGE_STIMULUS
2.59e-03 GO_DNA_REPAIR
2.40e-04 GO_DNA_RECOMBINATION
1.98e-04 GO_DOUBLE_STRAND_BREAK_REPAIR
2.73e-02 GO_G2_DNA_DAMAGE_CHECKPOINT
2.73e-02 GO_TELOMERE_CAPPING
3.00e-02 GO_NEGATIVE_REGULATION_OF_PRODUCTION_OF_MOLECULAR_MEDIATOR_OF_IMMUNE_RESPONSE
2.73e-02 GO_REGULATION_OF_CELLULAR_SENESCENCE
1.89e-02 GO_APOPTOTIC_SIGNALING_PATHWAY
3.17e-03 GO_DNA_INTEGRITY_CHECKPOINT
6.22e-03 GO_CELL_CYCLE_CHECKPOINT
1.24e-03 GO_NON_RECOMBINATIONAL_REPAIR
1.48e-02 GO_EMBRYO_DEVELOPMENT_ENDING_IN_BIRTH_OR_EGG_HATCHING
4.18e-03 GO_RESPONSE_TO_ENDOGENOUS_STIMULUS
5.29e-03 GO_RESPONSE_TO_HORMONE
1.76e-02 GO_LIPID_LOCALIZATION
2.83e-02 GO_REGULATION_OF_LIPID_METABOLIC_PROCESS
2.99e-02 GO_GLYCEROLIPID_METABOLIC_PROCESS
2.73e-02 GO_SPERM_MOTILITY
3.00e-02 GO_GLYCEROLIPID_CATABOLIC_PROCESS
2.73e-02 GO_PROTEIN_LIPID_COMPLEX_ASSEMBLY
2.73e-02 GO_NEUTRAL_LIPID_CATABOLIC_PROCESS
2.73e-02 GO_BONE_GROWTH
2.73e-02 GO_ORGAN_MATURATION
3.00e-02 GO_ANTEROGRADE_AXONAL_TRANSPORT

e-figure 2: Gene-ontology Enrichment Analysis of Risk Gene Sets of BPD and Severe BPD.

GO analysis of genes (scored >2) that were selected in a combined burden test with NSV analysis of BPD vs. control (A), sBPD vs. mBPD + control (B), and sBPD vs. mBPD (C). Each subfigure depicts a cluster plot of genes selected by the 3 different burden tests (scored >2) in the enriched GO terms. The colored boxes represent genes belonging to GO terms. Genes and GO terms are re-clustered based on their presence in the binary matrix, and genes or GO terms with a similar pattern were grouped together. The P-value for GO enrichment is displayed in the plot, with a redder color indicating greater significance. The differential functional-enrichment test was performed using R packages (NetBID2, https://github.com/jyyulab/NetBID/releases), and we considered a biological process as defined in Gene Ontology. The software entailed hypergeometric testing to detect GO-term enrichment. The genome background was 3203 CES genes, and a p-value ≤ 0.1 was set as the threshold for significance.

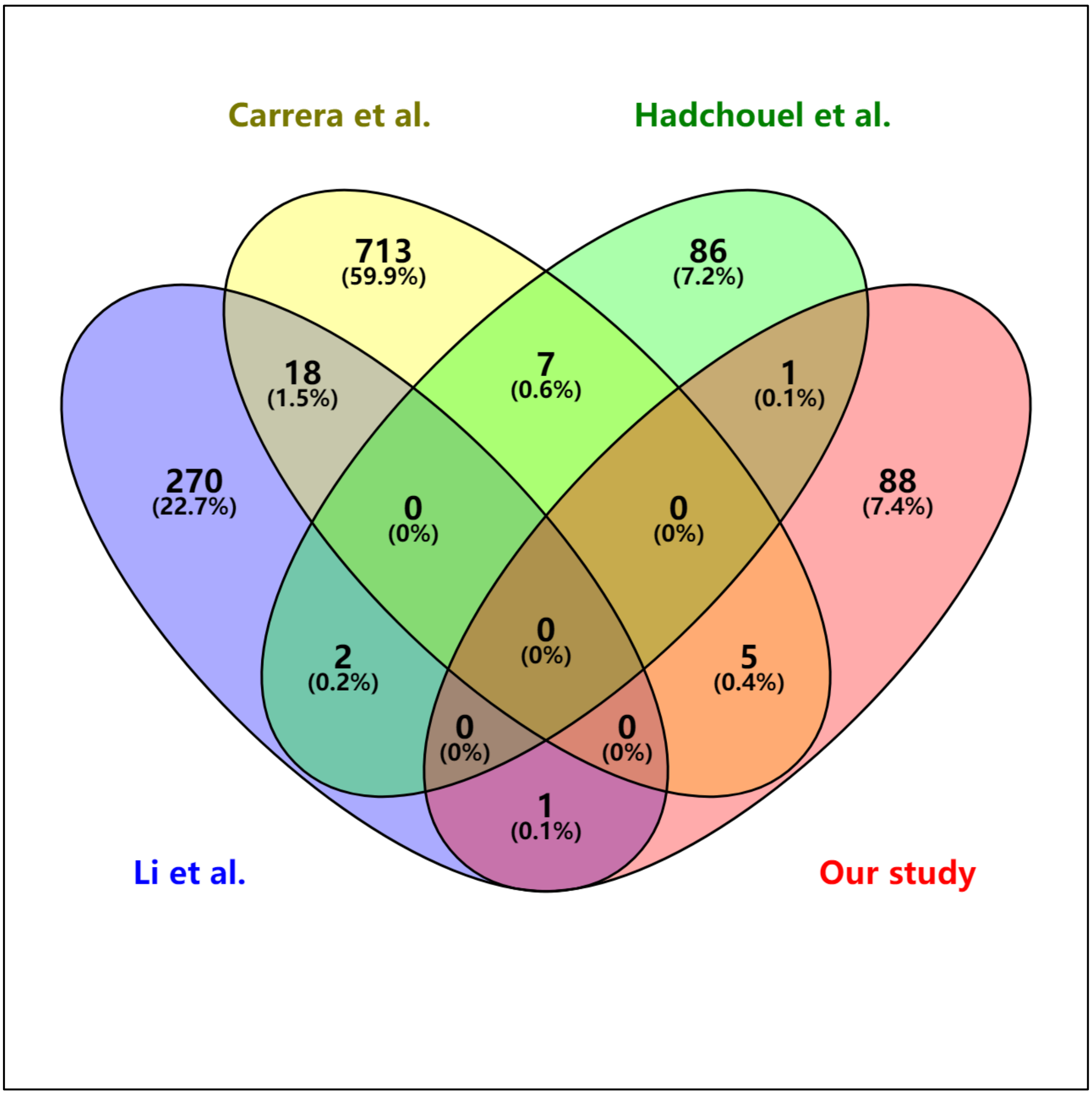

e-figure 3. Venn diagram of risk genes for BPD development reported in three previous studies [1,2,3] and ours.

1. Li J, Yu KH, Oehlert J, Jeliffe-Pawlowski LL, Gould JB, Stevenson DK, Snyder M, Shaw GM, O'Brodovich HM. Exome Sequencing of Neonatal Blood Spots and the Identification of Genes Implicated in Bronchopulmonary Dysplasia. Am J Respir Crit Care Med. 2015 Sep 1;192(5):589-96.

2. Carrera P, Di Resta C, Volonteri C, Castiglioni E, Bonfiglio S, Lazarevic D, Cittaro D, Stupka E, Ferrari M, Somaschini M; BPD and Genetics Study Group. Exome sequencing and pathway analysis for identification of genetic variability relevant for bronchopulmonary dysplasia (BPD) in preterm newborns: A pilot study. Clin Chim Acta. 2015 Dec 7;451(Pt A):39-45.

3. Hadchouel A, Decobert F, Besmond C, Delacourt C. Exome sequencing of extreme phenotypes in bronchopulmonary dysplasia. Eur J Pediatr. 2020 Apr;179(4):579-586.

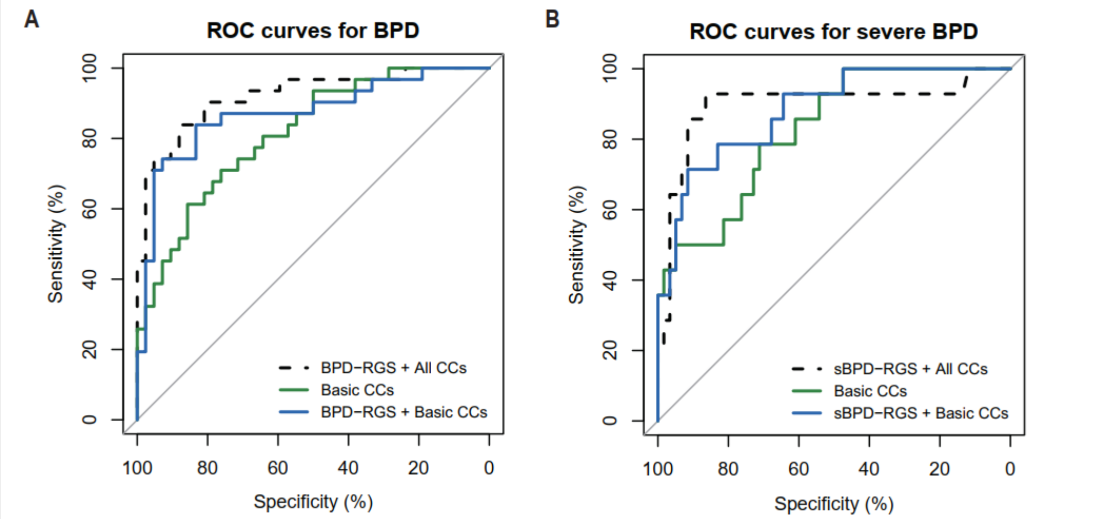

	AUC	95% CI	*P*	*P*
BPD−RGS + All CCs	0.922	0.857−0.987		Ref
Basic CCs	0.814	0.718−0.911	Ref	
BPD−RGS + Basic CCs	0.872	0.783−0.96	0.125	0.036

	AUC	95% CI	*P*	*P*
sBPD−RGS + All CCs	0.898	0.775−1		Ref
Basic CCs	0.827	0.714−0.94	Ref	
sBPD−RGS + Basic CCs	0.881	0.785−0.978	0.32	0.68

e-figure 4: Receiver Operating Characteristic (ROC) Analyses of Predictive Models for Infants with BPD or Severe BPD.

The comparisons of predictive models for BPD and sBPD. P values show the areas under the ROC curves (AUCs) for the 3 different models. (A) ROC analyses of the prediction of BPD by the combination of BPD-RGS and all clinical characteristics model, the basic clinical characteristics model, and the combined BPD-RGS and basic clinical characteristics model. (B) ROC analyses of the prediction of severe BPD by the combined sBPD-RGS and all clinical characteristics model, the basic clinical characteristics model, and the combined sBPD-RGS and basic clinical characteristics model.

The RGS here is the set of genes found by potentially deleterious rare variants association analysis (e-Table 4). RGS: risk gene set; CCs: clinical characteristics.

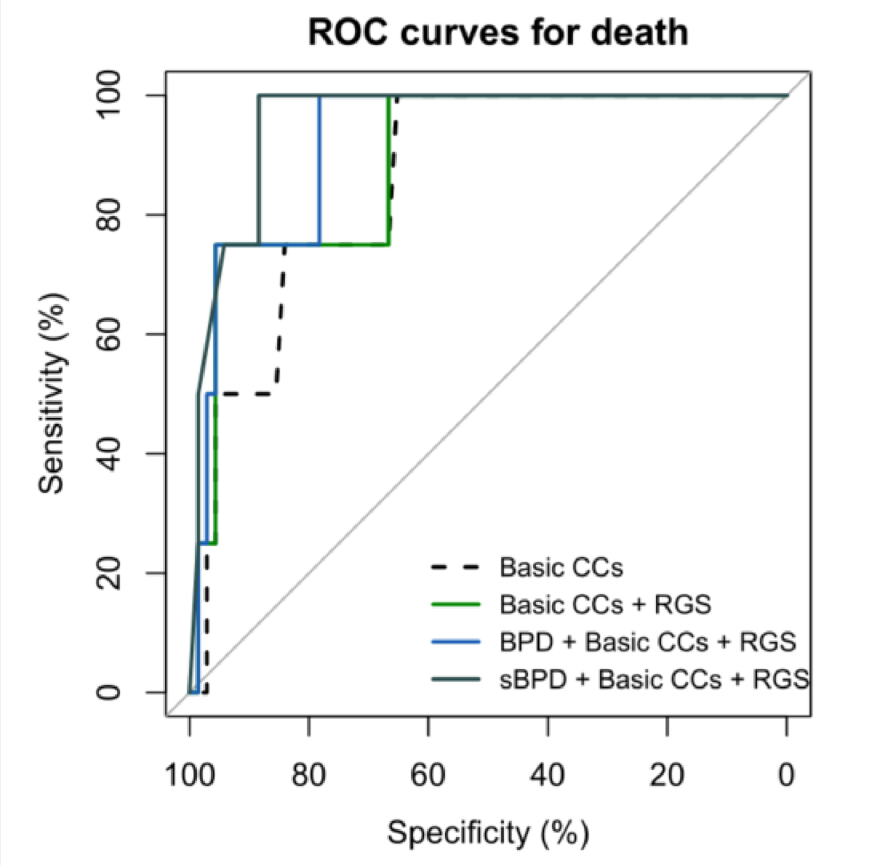

	AUC	95% CI	*P*	*P*
Basic CCs	0.859	0.707−1	Ref	
Basic CCs + RGS	0.891	0.737−1	0.258	Ref
BPD + Basic CCs + RGS	0.924	0.822−1		0.274
sBPD + Basic CCs + RGS	0.957	0.898−1		0.221

e-figure 5: Feature's Coefficient in Predictive Models for Infant Deaths with BPD. The comparisons of predictive models for infant deaths with BPD. P values show the areas under the ROC curves (AUCs) of the 3 different models. Basic CCs included birth weight, gestational age, and invasive mechanical ventilation. The dotted line portrays the ROC analysis of the prediction of deaths purely by basic CCs. The red line combines RGS and basic CCs. The blue line combines RGS, basic CCs, and the presence of BPD. The black line combines RGS, basic CCs, and the presence of sBPD. RGS: risk gene set; CCs: clinical characteristics.

e-Table 1 Maternal and neonatal characteristics of 245 premature infants.

Characteristic	Total infants (n=245)	Mild BPD (n=44)	Moderate BPD (n=20)	Severe BPD (n=67)	p-value	BPD (n=131)	No BPD (n=114)	p-value
Gestational age (wk, median [IQR])	29.1 (27.9-30.6)	29 (27.8-29.7)	28.8 (27.5-30.6)	27.6 (26.6-29.4)	<0.001	28.1 (27.1-29.8)	30 (28.6-31.1)	<0.001
Birth weight (gr, median [IQR])	1200 (1020-1410)	1160 (1072.5-1280)	1110 (1017.5-1271.3)	1035 (915-1200)	<0.001	1090 (945-1260)	1332.5 (1131.3-1570)	<0.001
Sex (male), No. (%)	140 (57.1%)	26 (59.1%)	13 (65%)	41 (61.2%)	0.579	80 (61.1%)	60 (52.6%)	0.229
In-vitro fertilization, No. (%)	77 (31.4%)	19 (43.2%)	6 (30%)	25 (37.3%)	0.069	50 (38.2%)	27 (23.7%)	0.022
Multiple births, No. (%)	117 (47.8%)	27 (61.4%)	13 (65%)	32 (47.8%)	0.032	72 (55.0%)	45 (39.5%)	0.022
Prenatal dexamethasone treatment, No. (%)	170 (69.4%)	34 (77.3%)	14 (70%)	47 (70.1%)	0.571	95 (72.5%)	75 (65.8%)	0.317
Intravenous glucocorticoid therapy, No. (%)	26 (10.6%)	2 (4.5%)	1 (5%)	22 (32.8%)	<0.001	25 (19.1%)	1 (0.9%)	<0.001
Caffeine therapy, No. (%)	197 (80.4%)	40 (90.9%)	19 (95%)	57 (85.1%)	0.005	116 (88.5%)	81 (71.1%)	0.001
Premature rupture of membranes, No. (%)	107 (43.7%)	21 (47.7%)	1 (5%)	22 (32.8%)	0.214	52 (39.7%)	55 (48.2%)	0.224
Placental abruption, No. (%)	10 (4.1%)	0	0	5 (7.5%)	0.265	6 (4.6%)	4 (3.5%)	0.921
Fetal distress, No. (%)	20 (8.2%)	5 (11.4%)	0	3 (4.5%)	0.218	8 (6.1%)	12 (10.5%)	0.305
Intrauterine growth retardation, No. (%)	8 (3.3%)	2 (4.5%)	0	3 (4.5%)	0.715	5 (3.8%)	3 (2.6%)	0.873
Pregnancy-induced hypertension, No. (%)	32 (13.1%)	9 (20.5%)	1 (5%)	9 (13.4%)	0.158	18 (13.7%)	14 (12.3%)	0.882
Eclampsia or preeclampsia, No. (%)	28 (11.4%)	7 (15.9%)	2 (10%)	8 (11.9%)	0.615	16 (12.2%)	12 (10.5%)	0.831
Gestational diabetes, No. (%)	39 (15.9%)	5 (11.4%)	2 (10%)	12 (17.9%)	0.653	19 (14.5%)	20 (17.5%)	0.636
Gestational hypothyroidism, No. (%)	13 (5.3%)	2 (4.5%)	4 (20%)	3 (4.5%)	0.797	7 (5.3%)	6 (5.3%)	1.000
Abnormal amniotic fluid, No. (%)	50 (20.4%)	6 (13.6%)	16 (80%)	14 (20.9%)	0.646	24 (18.3%)	26 (22.8%)	0.478
Hypotonia, No. (%)	173 (70.6%)	34 (77.3%)	18 (90%)	45 (67.2%)	0.487	95 (72.5%)	78 (68.4%)	0.574
Neonatal respiratory distress syndrome, No. (%)	194 (79.2%)	40 (90.9%)	19 (95%)	55 (82.1%)	0.019	113 (86.3%)	81 (71.1%)	0.006
Postnatal resuscitation, No. (%)	224 (91.4%)	43 (97.7%)	17 (85%)	64 (95.5%)	0.040	126 (96.2%)	98 (86.0%)	0.009
Invasive mechanical ventilation, No. (%)	142(58.0%)	24 (54.5%)	11 (55%)	61 (91%)	<0.001	102 (77.9%)	40 (35.1%)	<0.001
Invasive mechanical ventilation ⩾ 7 d, No. (%)	79 (32.2%)	10 (22.7%)	19 (95%)	51 (76.1%)	<0.001	72 (55.0%)	7 (6.1%)	<0.001
Patent ductus arteriosus, No. (%)	205 (83.7%)	40 (90.9%)	20 (100%)	59 (88.1%)	0.029	118 (90.1%)	87 (76.3%)	0.006
Atrial septal defect (ASD) or Ventricular septal defect (VSD), No.	63 (25.7%)	6 (13.6%)	17 (85%)	26 (38.8%)	0.001	41 (31.3%)	22 (19.3%)	0.046

(%)								
Respiratory failure, No. (%)	229 (93.5%)	44 (100.0%)	1 (5%)	67 (100%)	<0.001	131 (100.0%)	98 (86.0%)	<0.001
Thrombocytopenia, No. (%)	27 (11.0%)	2 (4.5%)	5 (25%)	17 (25.4%)	<0.001	23 (17.6%)	4 (3.5%)	0.001
Hypothyroidism, No. (%)	35 (14.3%)	7 (15.9%)	9 (45%)	10 (14.9%)	0.935	19 (14.5%)	16 (14%)	1.000
Coagulation disorders, No. (%)	73 (29.8%)	10 (22.7%)	4 (20%)	33 (49.3%)	<0.001	52 (39.7%)	21 (18.4%)	<0.001
Intraventricular hemorrhage, No. (%)	75 (30.6%)	13 (29.5%)	1 (5%)	32 (47.8%)	<0.001	55 (42.0%)	20 (17.5%)	<0.001
Death, No. (%)	19 (7.8%)	0	16 (80%)	19 (28.4%)	<0.001	19 (14.5%)	0	<0.001
Airway malformations, No. (%)	11 (4.5%)	1 (2.3%)	13 (65%)	8 (11.9%)	0.005	10 (7.6%)	1 (0.9%)	0.025
Early onset infections, No. (%)	194 (79.2%)	35 (79.5%)	13 (65%)	63 (94.0%)	0.002	114 (87.0%)	80 (70.2%)	0.002
Late onset infections, No. (%)	91 (37.1%)	14 (31.8%)	6 (30%)	33 (49.3%)	0.001	60 (45.8%)	31 (27.2%)	0.004

All summary data are medians (25%-75% percentile) or counts (%). wk: week; gr: gram.

e-Table 2 Genes with a significant burden for LOF/MIS variants.

Comparison	Gene	Samples (n)	Case (n) LOF	Case (n) MIS	Control (n) LOF	Control (n) MIS	Control (n)	Case (n)	LOF p-value	MIS p-value	NSV (n)	Score
BPD vs control	*OBSL1*	31	11	18	0	13	114	131	< 0.001	0.362	1	7.600
BPD vs control	*NTRK1*	34	1	15	1	2	114	131	0.785	0.002	2	4.884
BPD vs control	*CHRNA4*	10	0	10	1	0	114	131	1	0.002	1	3.791
BPD vs control	*PDE11A*	24	7	14	2	3	114	131	0.124	0.011	0	3.766
BPD vs control	*FRG1*	24	9	10	2	3	114	131	0.049	0.07	0	3.764
BPD vs control	*SPTAN1*	26	0	19	0	4	114	131	1	0.002	1	3.613
BPD vs control	*DCC*	17	1	12	0	2	114	131	0.535	0.011	1	3.507
BPD vs control	*BDP1*	14	3	10	0	2	114	131	0.151	0.03	0	3.159
BPD vs control	*C5*	10	1	7	1	0	114	131	0.785	0.012	1	3.147
BPD vs control	*AGRN*	39	0	35	0	16	114	131	1	0.011	1	2.971
BPD vs control	*AGXT*	60	0	7	0	0	114	131	1	0.012	1	2.937
BPD vs control	*TSHZ1*	7	0	7	0	0	114	131	1	0.012	1	2.937
BPD vs control	*COL1A2*	57	0	31	0	10	114	131	1	0.001	0	2.871
BPD vs control	*TERT*	11	0	10	0	0	114	131	1	0.002	0	2.791
BPD vs control	*DDHD1*	9	0	9	0	1	114	131	1	0.017	1	2.776
BPD vs control	*PTPRQ*	27	0	15	0	4	114	131	1	0.017	1	2.774
BPD vs control	*PEX12*	6	0	6	0	0	114	131	1	0.022	1	2.655

BPD vs control	*SACS*	30	0	27	0	12	114	131	1	0.023	1	2.637
BPD vs control	*CLCN1*	21	0	12	1	3	114	131	1	0.029	1	2.540
BPD vs control	*GAA*	30	0	8	0	1	114	131	1	0.029	1	2.534
BPD vs control	*VPS13A*	32	0	26	0	12	114	131	1	0.032	1	2.490
BPD vs control	*NPHS1*	54	1	41	0	21	114	131	0.535	0.015	0	2.376
BPD vs control	*GIGYF2*	17	0	5	0	0	114	131	1	0.042	1	2.375
BPD vs control	*VSX2*	5	0	5	0	0	114	131	1	0.042	1	2.375
BPD vs control	*LAMB2*	28	0	22	0	10	114	131	1	0.046	1	2.333
BPD vs control	*DMPK*	17	4	7	4	0	114	131	0.713	0.012	0	2.231
BPD vs control	*NDUFS7*	9	0	8	0	0	114	131	1	0.006	0	2.220
BPD vs control	*CENPJ*	22	0	19	0	5	114	131	1	0.006	0	2.210
BPD vs control	*DHTKD1*	8	1	6	0	0	114	131	0.535	0.022	0	2.199
BPD vs control	*TSC2*	35	1	12	3	2	114	131	0.954	0.011	0	2.004
sBPD vs mBPD	*DOCK6*	21	0	15	0	3	64	67	1	0.003	1	3.545
sBPD vs mBPD	*ABCA3*	24	0	12	0	2	64	67	1	0.006	1	3.249
sBPD vs mBPD	*FGFR3*	17	0	12	0	1	64	67	1	0.001	0	2.851
sBPD vs mBPD	*SPG7*	20	0	8	0	1	64	67	1	0.019	1	2.711
sBPD vs mBPD	*PRKDC*	21	0	9	0	2	64	67	1	0.033	1	2.487
sBPD vs mBPD	*APOB*	29	0	15	0	6	64	67	1	0.036	1	2.450
sBPD vs mBPD	*RBBP8*	9	0	7	0	1	64	67	1	0.036	1	2.444
sBPD vs mBPD	*ZFYVE26*	12	0	7	1	1	64	67	1	0.036	1	2.444
sBPD vs mBPD	*DIP2B*	12	0	7	0	0	64	67	1	0.008	0	2.108
sBPD vs mBPD/control	*ACADSB*	10	5	1	3	6	178	67	0.037	0.897	2	4.907
sBPD vs mBPD/control	*TCIRG1*	15	3	5	0	13	178	67	0.02	0.578	1	4.646
sBPD vs mBPD/control	*OBSL1*	31	7	10	4	21	178	67	0.011	0.323	0	4.408
sBPD vs mBPD/control	*FGFR3*	17	0	12	0	9	178	67	1	0.003	1	3.594
sBPD vs mBPD/control	*BDP1*	14	3	2	0	10	178	67	0.02	0.887	0	3.459
sBPD vs mBPD/control	*RBBP8*	9	0	7	0	3	178	67	1	0.005	1	3.290
sBPD vs mBPD/control	*SPG7*	20	0	8	1	5	178	67	1	0.008	1	3.077
sBPD vs mBPD/control	*GNAS*	17	0	11	0	6	178	67	1	< 0.001	0	3.028
sBPD vs mBPD/control	*ELP2*	11	0	5	0	0	178	67	1	0.001	0	2.864
sBPD vs mBPD/control	*POMT1*	8	1	3	0	0	178	67	0.273	0.02	0	2.830
sBPD vs mBPD/control	*MKKS*	8	0	7	0	5	178	67	1	0.021	1	2.686

sBPD vs mBPD/control	*CLCN1*	21	0	8	1	7	178	67	1	0.025	1	2.594
sBPD vs mBPD/control	*DDHD1*	9	0	6	0	4	178	67	1	0.028	1	2.557
sBPD vs mBPD/control	*HPS4*	10	0	6	0	4	178	67	1	0.028	1	2.557
sBPD vs mBPD/control	*EFHC1*	5	0	4	1	2	178	67	1	0.049	1	2.308
sBPD vs mBPD/control	*IL4R*	4	0	4	0	2	178	67	1	0.049	1	2.308
sBPD vs mBPD/control	*PTF1A*	10	0	4	0	2	178	67	1	0.049	1	2.308
sBPD vs mBPD/control	*CCT5*	11	0	7	0	3	178	67	1	0.005	0	2.290
sBPD vs mBPD/control	*CFD*	6	0	4	0	0	178	67	1	0.005	0	2.281
sBPD vs mBPD/control	*NDUFS7*	9	0	6	0	2	178	67	1	0.006	0	2.219
sBPD vs mBPD/control	*PALB2*	15	0	10	0	8	178	67	1	0.008	0	2.085

LOF/MIS: loss of function variants and missense variants; sBPD: severe BPD; mBPD: mild BPD and moderate BPD.

e-Table 3 Genes with a significant burden for dLOF/dMIS variants.

Comparison	Gene	Samples (n)	Case (n) dLOF	Case (n) dMIS	Control (n) dLOF	Control (n) dMIS	Control (n)	Case (n)	dLOF p-value	dMIS p-value	NSV (n)	Score
BPD vs control	*OBSL1*	28	11	15	0	9	114	131	< 0.001	0.237	2	8.784
BPD vs control	*MSH2*	24	21	3	8	2	114	131	0.023	0.565	1	4.543
BPD vs control	*DMPK*	15	6	7	1	0	114	131	0.085	0.012	0	4.073
BPD vs control	*SMPD1*	34	32	1	17	3	114	131	0.044	0.954	1	3.731
BPD vs control	*DCTN1*	8	0	8	0	1	114	131	1.000	0.029	2	3.534
BPD vs control	*NTRK1*	15	1	13	1	2	114	131	0.785	0.006	1	3.406
BPD vs control	*DCC*	13	1	11	0	2	114	131	0.535	0.018	1	3.282
BPD vs control	*NPHS1*	42	1	36	0	14	114	131	0.535	0.002	0	3.167
BPD vs control	*PDE11A*	18	7	11	2	3	114	131	0.124	0.045	0	3.153
BPD vs control	*DDX11*	10	1	9	0	2	114	131	0.535	0.049	1	2.850
BPD vs control	*VPS13A*	23	0	23	0	6	114	131	1	0.002	0	2.655
BPD vs control	*DDHD1*	6	0	6	0	0	114	131	1	0.022	1	2.655
BPD vs control	*TSHZ1*	6	0	6	0	0	114	131	1	0.022	1	2.655
BPD vs control	*LRBA*	28	0	20	0	8	114	131	1	0.033	1	2.485
BPD vs control	*GOT1*	8	1	7	0	0	114	131	0.535	0.012	0	2.480
BPD vs control	*MTTP*	9	2	5	0	0	114	131	0.285	0.042	0	2.466
BPD vs control	*KRT10*	13	2	11	0	3	114	131	0.285	0.045	0	2.433
BPD vs control	*LAMB2*	22	0	21	0	9	114	131	1	0.039	1	2.404

BPD vs control	*CHRNA4*	5	0	5	1	0	114	131	1	0.042	1	2.375
BPD vs control	*GIGYF2*	5	0	5	0	0	114	131	1	0.042	1	2.375
BPD vs control	*PEX12*	5	0	5	0	0	114	131	1	0.042	1	2.375
BPD vs control	*TNFRSF11B*	5	0	5	0	0	114	131	1	0.042	1	2.375
BPD vs control	*CLCN1*	12	0	11	1	3	114	131	1	0.045	1	2.342
BPD vs control	*TSC2*	13	1	11	3	1	114	131	0.954	0.005	0	2.316
BPD vs control	*TMPRSS6*	11	0	11	0	1	114	131	1	0.005	0	2.276
BPD vs control	*ACAN*	31	0	28	0	11	114	131	1	0.009	0	2.035
BPD vs control	*SPTAN1*	11	0	10	0	1	114	131	1	0.009	0	2.024
sBPD vs mBPD	*DOCK6*	12	0	11	0	1	64	67	1	0.003	1	3.556
sBPD vs mBPD	*ACAN*	31	0	19	0	9	64	67	1	0.037	2	3.436
sBPD vs mBPD	*FGFR3*	13	0	12	0	1	64	67	1	0.001	0	2.851
sBPD vs mBPD	*ATM*	7	0	5	2	0	64	67	1	0.032	1	2.489
sBPD vs mBPD	*ZFYVE26*	6	0	5	1	0	64	67	1	0.032	1	2.489
sBPD vs mBPD	*LMF1*	17	0	12	0	4	64	67	1	0.037	1	2.435
sBPD vs mBPD	*LOXHD1*	16	1	11	0	3	64	67	0.511	0.028	0	2.143
sBPD vs mBPD/control	*COL3A1*	18	2	11	0	10	178	67	0.074	0.010	1	5.271
sBPD vs mBPD/control	*ACADSB*	10	5	1	3	6	178	67	0.037	0.897	2	4.907
sBPD vs mBPD/control	*ACAN*	31	0	19	0	20	178	67	1	0.002	2	4.808
sBPD vs mBPD/control	*OBSL1*	28	7	7	4	17	178	67	0.011	0.500	0	4.218
sBPD vs mBPD/control	*FMO3*	11	3	5	1	2	178	67	0.063	0.018	0	4.147
sBPD vs mBPD/control	*TCIRG1*	8	3	4	0	5	178	67	0.020	0.209	0	4.088
sBPD vs mBPD/control	*DMPK*	15	5	3	2	4	178	67	0.018	0.292	0	4.038
sBPD vs mBPD/control	*DOCK6*	12	0	11	0	11	178	67	1	0.015	2	3.817
sBPD vs mBPD/control	*FGFR3*	13	0	12	0	9	178	67	1	0.003	1	3.594
sBPD vs mBPD/control	*BDP1*	6	3	0	0	4	178	67	0.020	1	0	3.407
sBPD vs mBPD/control	*LRBA*	28	0	14	0	14	178	67	1	0.006	1	3.243
sBPD vs mBPD/control	*IL17RA*	4	0	4	0	1	178	67	1	0.021	1	2.684
sBPD vs mBPD/control	*LMF1*	17	0	12	0	14	178	67	1	0.024	1	2.623
sBPD vs mBPD/control	*TG*	28	0	18	0	28	178	67	1	0.038	1	2.421
sBPD vs mBPD/control	*DDHD1*	6	0	4	0	2	178	67	1	0.049	1	2.308
sBPD vs mBPD/control	*TSHZ1*	6	0	4	0	2	178	67	1	0.049	1	2.308
sBPD vs mBPD/control	*ABCA1*	8	0	7	0	3	178	67	1	0.005	0	2.290

sBPD vs mBPD/control	*CCT5*	9	0	7	0	3	178	67	1	0.005	0	2.290
sBPD vs mBPD/control	*ELP2*	4	0	4	0	0	178	67	1	0.005	0	2.281
sBPD vs mBPD/control	*WAC*	4	0	4	0	0	178	67	1	0.005	0	2.281
sBPD vs mBPD/control	*PRF1*	9	0	6	0	2	178	67	1	0.006	0	2.219

dLOF/dMIS: predicted potentially deleterious or deleterious loss of function variants and missense variants; sBPD: severe BPD; mBPD: mild BPD and moderate BPD.
